# Editorial: Signaling crosstalk in obesity and adipose tissue-derived metabolic diseases

**DOI:** 10.3389/fphys.2023.1233284

**Published:** 2023-06-12

**Authors:** Lei Huang

**Affiliations:** Department of Molecular, Cell and Cancer Biology, University of Massachusetts Chan Medical School, Worcester, MA, United States

**Keywords:** obesity, diabetes, metabolic disorders, thermogenesis, white fat browning

Adipose tissue, comprising white adipose tissue (WAT) and brown adipose tissue (BAT), plays a crucial role in maintaining glucose balance and energy equilibrium. WAT stores excess energy, while BAT generates heat through non-shivering thermogenesis (Harms and Seale, 2013; Huang et al., 2017). Certain stimuli can convert WAT into brown-like adipocytes, known as beige fat. Both BAT and beige fat activity decrease with body mass index and age (Cypess et al., 2009; Bartelt and Heeren, 2014), making them potential targets for obesity and metabolic diseases. However, regulating adipose thermogenesis is essential to prevent wasteful energy expenditure in situations (Huang et al., 2022), like limited food, cancer cachexia, burns, hyperthyroidism, and infection (Nedergaard and Cannon, 2014; Abdullahi and Jeschke, 2016; Petruzzelli and Wagner, 2016; Daas et al., 2018). Despite extensive research on thermogenesis mechanisms, many genes and regulatory networks remain undiscovered. Our Research Topic explored metabolic signaling, genes, and potential therapies for obesity and adipose-related metabolic disorders, featuring reviews, original research, and a brief report.

Adipose tissue performs crucial functions in energy metabolism and maintaining the body’s metabolic balance, which involves the release of signaling molecules called adipokines. Neuregulin 4 (Nrg4), a recently discovered adipokine primarily released by BAT plays a significant role in managing energy balance and regulating glucose and lipid metabolism (Pfeifer, 2015). Nrg4 enhances thermogenesis in BAT by increasing sympathetic innervation, thereby regulating energy expenditure. This function potentially has positive metabolic effects. Additionally, Nrg4 has been found to have beneficial impacts on metabolic dysfunctions associated with insulin resistance, obesity, non-alcoholic fatty liver disease, and diabetes. These effects are believed to occur through various mechanisms, including reducing inflammation, regulating autophagy, promoting angiogenesis, and normalizing lipid metabolism. However, research findings on the clinical effects of Nrg4 are not consistently conclusive, emphasizing the need for further investigation. Nonetheless, the potential protective effects of Nrg4 on metabolism make it a promising candidate for targeted therapies in treating metabolic disorders. Understanding the precise role and mechanisms of Nrg4 could lead to innovative therapeutic strategies in the future.

Intermittent fasting (IFR) has gained recognition for its potential to boost metabolism (Rosas Fernandez et al., 2023), although its exact mechanism remains unclear. One theory proposes that IFR may stimulate the formation of beige adipocytes, which possess thermogenic properties, within WAT. Research has revealed a potential role of the liver in this process, specifically through the release of pregnancy zone protein (PZP) during IFR. This coincides with the relocation of another protein, grp78, to the membranes of brown adipocytes, known for their calorie-burning capabilities. Consequently, BAT becomes activated, contributing to the metabolic benefits associated with IFR. The significance of PZP in metabolism is underscored by observations that mice lacking a functional liver do not exhibit the expected increase in energy expenditure during IFR. This suggests that liver-derived PZP may be instrumental in these metabolic enhancements. Further investigation using a mouse model that overproduces PZP in adipose tissue demonstrated improved metabolic disorders on a high-fat diet during IFR. This was accompanied by BAT activation, increased expression of UCP1 protein, angiogenesis in WAT, and upregulation of genes related to glucose utilization. These findings suggest that overexpressing PZP in adipose tissue enhances WAT’s ability to convert energy and indicates that WAT may be another direct target for PZP during IFR, besides BAT. Therefore, PZP could serve as a key molecular player in the metabolic benefits of IFR, linking the liver, BAT, and WAT in a coordinated response to fasting periods. However, further research is required to validate these findings and understand their implications for therapeutic strategies in metabolic disorders and obesity.

AMPK (AMP-activated protein kinase) is a crucial enzyme involved in maintaining cellular energy balance (Shackelford and Shaw, 2009). Its activation promotes glucose uptake and fatty acid oxidation, making it a promising target for treating T2D characterized by insulin resistance and impaired glucose metabolism. However, several questions remain regarding AMPK’s behavior in the presence of obesity. It is unclear if obesity affects AMPK expression or activity in adipocytes. Additionally, the study mentioned sheds light on AMPKβ isoforms, which could be potential targets for AMPK activators. The study suggests that both AMPKβ isoforms play significant roles in AMPK activity in human and mouse adipocytes, providing insights for therapeutic strategies targeting these isoforms. Interestingly, the dominance of these isoforms may vary depending on the species or cell type. Surprisingly, obesity does not appear to impact AMPK subunit expression or kinase activity in adipocytes from subcutaneous fat tissue across individuals with varying BMI. This finding suggests a complex interplay between AMPK, obesity, and T2D, warranting further exploration. Overall, this research enhances our understanding of AMPK in the context of T2D and obesity, calling for additional studies to elucidate the implications for treatment approaches.

Diet-induced obesity (DIO) in laboratory rodents is commonly used to study the underlying mechanisms of obesity (de Moura et al., 2021). To evaluate the therapeutic potential of natural products and bioactive compounds, rodents are fed obesogenic diets high in sugar and/or fat. However, these diets often lack detailed composition information, limiting our understanding of the diverse metabolic responses they elicit. This study aimed to address this limitation by characterizing and comparing the metabolic responses induced by three different diets in rats. Young male Wistar rats were assigned to each dietary group, and their metabolic responses were assessed after 17 weeks. Projection-based multivariate statistical models, such as principal component analysis (PCA) and orthogonal partial least squares-discriminant analysis (OPLS-DA), were utilized to explore associations between body composition and metabolism measures. The study identified distinct metabolic profiles associated with each diet. The medium-fat/high-sugar diet led to disruptions in insulin homeostasis and adipose tissue function, while the high-fat diet resulted in altered lipid and liver metabolism. These findings highlight the significant impact of dietary composition on metabolic and pathophysiological outcomes in rodent models of DIO. To further validate their findings, the researchers conducted a literature survey on rat dietary intervention studies, revealing that detailed dietary composition is often overlooked, limiting the mechanistic insights into DIO and its translation to human obesity. In conclusion, this study emphasizes the importance of considering dietary composition in DIO research. It provides valuable insights into the distinct metabolic responses induced by different obesogenic diets and emphasizes the need for improved reporting of dietary composition in future studies to enhance our understanding of DIO and its relevance to human obesity.

Non-alcoholic fatty liver disease (NAFLD) is a prevalent chronic liver condition associated with insulin resistance and metabolic syndrome, making it a significant health concern in developed countries (Powell et al., 2021). NAFLD encompasses a spectrum of conditions, ranging from simple steatosis to non-alcoholic steatohepatitis and cirrhosis. Monitoring the progression of fibrosis, characterized by excessive scar tissue in the liver, is vital for managing and predicting outcomes in NAFLD patients. However, liver biopsy, the current gold standard for fibrosis staging, has limitations due to its invasive nature, potential complications, sampling variability, and patient reluctance. To overcome these challenges, non-invasive alternatives have been developed and extensively studied. These include imaging-based techniques like transient elastography (FibroScan), magnetic resonance elastography (MRE), and ultrasound-based elastography, as well as serum biomarkers such as the Fibrosis-4 (FIB-4) index and NAFLD fibrosis score (NFS). This review provides an overview of these non-invasive methods, discussing their advantages, limitations, and diagnostic performance in assessing fibrosis in NAFLD. The aim is to assist clinicians and researchers in selecting appropriate tools for fibrosis staging, considering factors such as safety, convenience, and patient acceptance.
